# How far does the moral dilemma go? Neural and autonomic correlates contribute to explain emotional conflict and empathy behavior in Sophie’s Choice

**DOI:** 10.3389/fpsyg.2026.1764212

**Published:** 2026-09-09

**Authors:** Michela Balconi, Carlotta Acconito, Ruggero Eugeni, Federico Bionda, Katia Rovelli, Laura Angioletti, Flavia Ciminaghi

**Affiliations:** 1 International Research Center for Cognitive Applied Neuroscience (IrcCAN), Università Cattolica del Sacro Cuore, Milan, Italy; 2 Research Unit in Affective and Social Neuroscience, Department of Psychology, Università Cattolica del Sacro Cuore, Milan, Italy; 3 Department of Communication and Performing Arts, Università Cattolica del Sacro Cuore, Brescia, Italy

**Keywords:** dynamic paradigm, EEG, emotion, moral dilemma, skin conductance

## Abstract

**Introduction:**

This study examined EEG and autonomic responses during an emotionally intense and morally paradoxical film scene from Sophie’s Choice.

**Methods:**

The cinematic content was segmented into 56 segments based on characters’ facial expressions and categorized by emotional valence. EEG and autonomic analyses were conducted across the full scene and by character.

**Results:**

Results showed increased delta activity in left frontal areas during morally ambiguous segments, indicating anticipatory control; theta in left temporoparietal regions reflected empathic engagement with the narrative; gamma increased during the victim’s distress, signaling emotional resonance. Skin conductance rose during Nazi-related scenes, highlighting external threat salience.

**Discussion:**

Findings illustrate how film can elicit distinct neurophysiological patterns, offering a powerful lens to study moral and emotional processing in immersive, narrative-driven contexts. These findings should be considered preliminary and exploratory, offering initial evidence on neurophysiological dynamics during morally complex cinematic experiences.

## 1 Introduction

Moral and emotional dilemmas have become central in neuroscience and psychology because they expose individuals to conflicting cognitive and affective demands (Helion and Ochsner, 2018; Cassioli et al., 2023). The concept of Double Bind (DB) (Bateson, 1972) describes such paradoxes, defined by irreconcilable conflicts and the absence of satisfactory solutions, leaving the individual grappling with cognitive dissonance, moral ambiguity, and emotional distress (Côté, 2023; Chikwe et al., 2024). Such paradoxical contexts not only generate dissonance but also recruit neural circuits responsible for conflict monitoring and social cognition.

Social cognition and empathy are central in the processing of moral dilemmas. In particular, the interaction between emotional resonance and moral reasoning is mediated by neural circuits encompassing the anterior insula, the anterior cingulate cortex (ACC), and the temporoparietal junction (TPJ), which dynamically integrates emotional and contextual information (Decety and Lamm, 2007; Seeley et al., 2007). Negative contents appear to exert a disproportionate influence on attentional allocation and affective salience detection processes, as reflected in increased gamma oscillations (Luo et al., 2014). Also, in dilemmas involving high emotional salience or moral conflict, the Mirror Neuron System (MNS; Cattaneo and Rizzolatti, 2009) mediates a deeper level of affective resonance and perspective-taking, thereby amplifying both emotional arousal and cognitive strain (Molnar-Szakacs, 2011; Lamm and Majdandžić, 2015).

Dual-process theories propose that moral decision-making involves the interplay of two systems (Kahneman, 2011): a fast, intuitive system (System 1) and a slower, more deliberative system (System 2). Event-related potential (ERP) evidence indicates that empathy primarily modulates early, automatic neural responses, whereas moral evaluation of others’ actions engages later, controlled processes (Cenka et al., 2024; Zhang et al., 2025), reflecting the dynamic interplay between bottom-up emotional resonance and top-down cognitive control during moral judgment. DB scenarios challenge both systems: System 1 initiates automatic emotional reactions to perceived threat or suffering through subcortical and limbic regions, while System 2 attempts to impose cognitive control, engaging higher-order cortical regions such as the dorsolateral prefrontal cortex (DLPFC) and temporo-parietal junction (TPJ). This dual engagement places significant demands on neural resources, leading to what has been described as cognitive-emotional conflict (Wells, 2017).

Neural oscillations measured through encephalography (EEG) provide a dynamic framework for examining how the brain coordinates cognitive and affective responses. For instance, research evidence suggests that gamma oscillations reflect high-level neural synchronization across distributed networks, supporting the integration of emotionally salient and morally complex information (Knyazev et al., 2016; Cassioli et al., 2023). Beta oscillations are often associated with evaluative processes and sustained attention during decision-making (Engel and Fries, 2010), while theta band activity has been involved in conflict detection, cognitive control, and social reasoning (Cavanagh and Frank, 2014; Balconi et al., 2024), particularly in the left temporo-parietal region (Billeke et al., 2014; van Noordt et al., 2015; Tendler and Wagner, 2015). Greater theta power has been also associated with emotionally salient decision-making in the medial PFC (Patel and Dusi, 2025). Studies have shown that the left TPJ is activated when individuals must engage in perspective-taking, particularly in situations requiring the resolution of conflicting negative moral or social norms (Decety and Lamm, 2007). In addition, delta activity in the prefrontal cortex has been associated with anticipatory control, reflecting the brain’s preparation for ambiguous scenarios (Nobre and van Ede, 2018; Cervantes Constantino et al., 2021).

Alongside neural measures, autonomic indices provide essential insight into the embodied dimension of moral processing. Skin conductance response (SCR) is particularly sensitive to emotionally salient or threatening stimuli (Balconi et al., 2014; Balconi and Angioletti, 2024). In scenarios involving externalized aggression or dominance, SCR reliably increases, reflecting sympathetic arousal and the orienting of attention toward danger (Jacobs et al., 2024).

Films provide an effective medium to study moral and emotional dilemmas because they present immersive, multimodal narratives (Tian et al., 2017), eliciting complex affective states that involve both empathy and moral evaluation. The Sophie’s Choice (Pakula, 1982) scene represents a paradigmatic DB: Sophie is forced by a Nazi officer to choose which of her two children will be sent to death. In the present context, the DB is characterized by the presence of mutually incompatible demands, each associated with unavoidable negative consequences, and the absence of an acceptable alternative or escape. The Sophie’s Choice scenario exemplifies this structure: the protagonist is forced to choose between two equally unacceptable outcomes (the death of one of her children), while refusal to choose results in an even more severe consequence (the death of both). This configuration fulfills the core criteria of a Double Bind, as it imposes an irresolvable conflict that simultaneously engages emotional, moral, and cognitive processes. This narrative context provides an exceptional stimulus for studying neurophysiological responses to paradoxical moral dilemmas.

Building on this framework, the present pilot study combined EEG and autonomic measures to explore viewers responses to Sophie’s Choice scene. For the EEG signal, we focused on neurophysiological activity within the prefrontal and temporo-parietal regions of interest, identified as central to the cognitive-emotional conflict. Signals were captured using a low-density, wearable EEG device, which offers a balance between non-invasive, ecologically-valid measurement and the regional localization required by our framework. This allowed for an analysis of key EEG frequency bands (delta, theta, alpha, beta and gamma) and potential lateralization differences, comparing the left (AF7) and right (AF8) prefrontal regions, and the left (TP9) and right (TP10) temporo-parietal regions, to test their distinct functional significance in processing the moral dilemma. The following hypotheses were formulated: (i) increased prefrontal delta activity compared to temporo-parietal regions during emotionally salient segments, reflecting anticipatory control; (ii) increased theta activity in the left temporo-parietal area during emotionally charged segments, reflecting perspective-taking and integration of emotional and contextual information, (iii) negative and aversive content will elicit increased gamma activity, suggesting integration of emotionally salient information; (iv) SCR will be greater during Nazi-related segments compared to Sophie-related segments, reflecting external threat salience.

## 2 Methods

### 2.1 Sample

The study involved a sample of 16 healthy adults (9 male, Mean_*age*_ = 31.1, SD_*age*_ = 9.2). Relevant demographic data are detailed in Table 1.

**TABLE 1:** Sample’s demographics data.

Variable	Mean (SD)/*n*
Age (years)	31.1 (9.2)
Age range	23–60
Gender	9 males/7 females
Parental status	Childless = 16
Education field	Social Sciences & Humanities = 10 Mathematics & Economics = 2 Psychology & Education = 4
Years of education	17.3 (1.3)

An a priori power analysis was performed using G*Power (version 3.1.9.7; Faul et al., 2007) to estimate the required sample size for the planned repeated-measures within-subject ANOVA, assuming a large effect size (*f* = 0.40). The analysis indicated that a minimum of 15 participants would be required to achieve a statistical power of 0.80 at an alpha level of 0.05. A large effect size (*f* = 0.40) was assumed in the *a priori* power analysis based on conventional benchmarks (Cohen, 1988), given the absence of prior effect size estimates for this specific paradigm.

Exclusion criteria included neurological or psychiatric disorders, major depressive episodes, high stress, low cognitive functioning, or use of psychoactive medication. In addition, all participants were selected to have no prior exposure to the movie Sophie’s Choice. All participants had normal or corrected-to-normal vision.

The research complied with the Declaration of Helsinki, (2013) and followed the General Data Protection Regulation (GDPR, EU Reg. 2016/679). Ethical approval was provided by the Ethics Committee of the Department of Psychology, Catholic University of the Sacred Heart, Milan, Italy. All participants gave their informed consent to participate in the study.

### 2.2 Procedure

**FIGURE 1:**
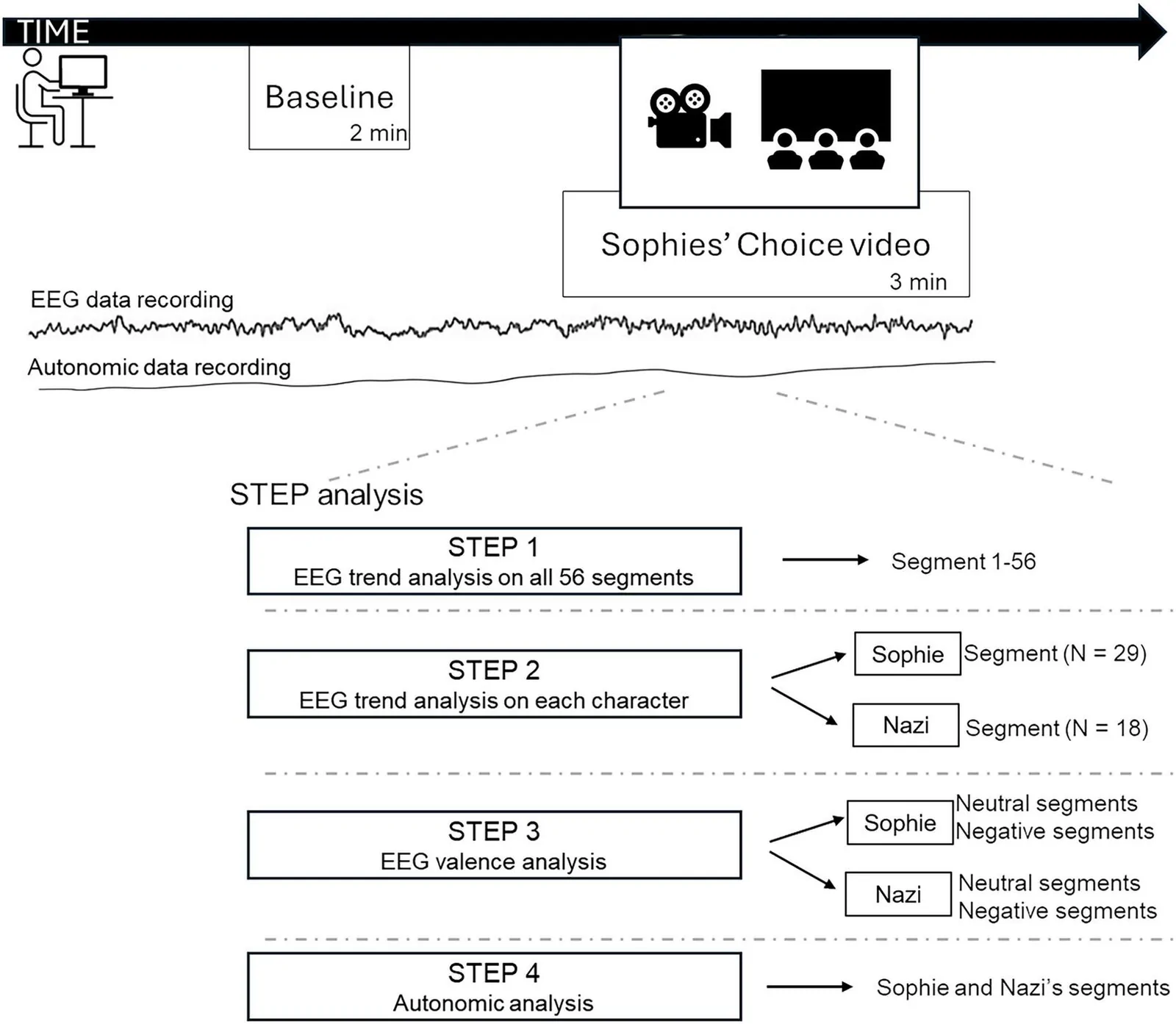
Procedure. Graphical description of the experimental procedure and different steps of statistical analysis.

Participants were seated comfortably in a quiet room, 80 cm from a monitor. After receiving instructions, they completed a 2-min resting-state baseline while EEG and autonomic signals were recorded. They then viewed a ∼3-min excerpt from Sophie’s Choice, in which Sophie is coerced by a Nazi officer to choose between her two children. This sequence was selected as a paradigmatic DB stimulus due to its emotional and moral salience. Participants were instructed to minimize movements to reduce artifacts in the signals. They did not receive any prior information about the content of the scene or background details about the movie. This choice was made to avoid expectation-driven biases and to ensure spontaneous and ecologically valid responses to the unfolding stimuli. Figure 1 describes the experimental procedure and the different steps of the data analysis.

### 2.3 Data acquisition and processing

Neurophysiological data were recorded using a Muse™ headband (version 2, InteraXon Inc., Toronto), which includes four active electrodes (AF7, AF8, TP9, TP10) following the 10–20 system, with a reference electrode placed at FPz. Data was sampled at 256 Hz and transferred via Bluetooth using the Mind Monitor application. A 50 Hz notch filter was automatically applied by the Muse hardware to reduce line noise. No other filter was applied. Mind Monitor software automatically estimates the Power Spectral Density (PSD) from the raw EEG signal using the device’s built-in Fast Fourier Transformation algorithm for the five standard bands (delta: 1–4 Hz, theta: 4–8 Hz, alpha: 7.5–13 Hz, beta: 13–30 Hz, and gamma: 30–44 Hz). All EEG PSD readings evaluated by Mind Monitor are typically in the −1:+1 range. The removal of artifacts (such as eye blinks, jaw clenching, and movements) was done through visual inspection of all the data. Finally, EEG activity collected during the Sophie’s Choice video was weighted by baseline values. Baseline weighting was applied by calculating the relative change from baseline for each segment, using the formula (task − baseline)/baseline. This produces both positive and negative values, reflecting increases or decreases in power relative to baseline, respectively.

Autonomic physiological data was collected using X-pert2000 portable Biofeedback system (Schuhfried GmbH, Austria). Skin conductance level (SCL) and response (SCR) were recorded via electrodes placed on the non-dominant index finger, while heart rate (HR) and heart rate variability (HRV) were measured using photoplethysmography. An accelerometer was used to monitor motion artifacts. Tonic SCL was quantified as the mean level per segment (μS), while SCRs were identified as peak amplitudes relative to tonic SCL and summarized as the mean amplitude per segment (μS). Signals were filtered with a 10 Hz low-pass IIR filter (EDA) and 35 Hz (ECG). HRV was quantified using Standard Deviation of Normal-to-Normal (NN) intervals (SDNN), a standard time-domain measure capturing overall heart rate variability. As for EEG, values were weighted over baseline using the same formula.

### 2.4 Data analysis

#### 2.4.1 Video segmentation

Before proceeding with the statistical analysis, the video was segmented into emotional segments by two independent judges. A total of 56 short segments (mean duration = 3.6 s) were first identified based on changes in facial expressions, with agreement between the two judges. Subsequently, the judges re-evaluated the 56 segments individually, using Ekman’s model of the seven primary emotions (Ekman and Keltner, 1997), and assigned an emotional label to each segment. Finally, the labels were compared and classified by emotional valence, resulting in three categories: positive, negative and neutral segments. An inter-judge agreement of 90% was achieved, and disagreements were resolved through discussion until full consensus was reached. However, for 7 segments no agreement was achieved (in terms of valence and belonging to a specific character); these segments, together with 2 additional segments depicting other characters (e.g., children), were excluded from the valence and character-specific trend analyses, but retained in the overall trend analysis including all 56 segments. For autonomic analysis, given the slower dynamics of skin conductance and cardiovascular measures, segments were merged into 25 longer segments (≥5 s) categorized as positive, negative, or neutral for Sophie and the Nazi separately.

#### 2.4.2 Statistical analysis

Neurophysiological data were analyzed in three steps:

First, an EEG trend analysis on all segments (56) was performed through a repeated-measure ANOVA with Electrodes (4: AF7, AF8, TP9, TP10) × Segment (56) as within-subject factors, separately for each frequency band.

Second, to explore the impact of each character on the neurophysiological responses of the viewers, two additional EEG trend analysis on each character were performed. Separate repeated-measures ANOVAs were computed for Sophie-related (29 segments) and Nazi-related (18 segments) segments, with Electrodes (4: AF7, AF8, TP9, TP10) and Segment (29 and 18, respectively) as within-subject independent variable for the five frequency bands.

The third step was an EEG valence analysis, performed to explore if the different emotional valence of the segment impacts the viewer on a neurophysiological level. As segments classified as positive were rare for both characters (*N* = 3 for Sophie and *N* = 4 for the Nazi), only neutral vs. negative segments were compared. Two separate repeated-measure ANOVAs with Emotion (2: Neutral, Negative) × Electrode (4: AF7, AF8, TP9, TP10) were performed for each character (Sophie and Nazi), for the five different frequency bands as dependent measures.

Although EEG data were recorded from 16 participants, the effective sample size varied slightly across analyses and frequency bands due to data cleaning procedures. After visual inspection and artifact rejection (average of 2% due to motion artifacts), a conservative approach was applied to remove statistical outliers and exclude incomplete cases (listwise deletion of participants performed by the statistical software). As a result, the effective sample size across analyses ranged between 14 and 16 participants, depending on the frequency band and specific analysis. These variations reflect the conservative preprocessing approach adopted to ensure the reliability of EEG signal quality across all conditions.

The fourth step of analysis involved autonomic data, analyzed with a repeated-measures ANOVA including Character (2: Sophie, Nazi) as within-subject factor and four indices (SCL, SCR, HR, HRV) as dependent variables. Due to the limited number of segments for each character a separate analysis was not performed. Some autonomic signals were also excluded due to poor signal quality or missing data due to technical issues (e.g., signal loss during acquisition), leading to different effective sample sizes across indices: SCL (*N* = 9), SCR (*N* = 7), HR (*N* = 13), and HRV (*N* = 11). These exclusions were performed to ensure the highest possible integrity and reliability of the autonomic data, even at the cost of a higher data reduction compared to EEG signals.

For all analyses, Greenhouse–Geisser correction was applied when the assumption of sphericity was violated, as assessed by Mauchly’s test. Bonferroni correction was used for multiple comparisons, and effect sizes were reported as eta squared (η^2^). Statistical significance was set at α = 0.05. The normality of data was evaluated using skewness and kurtosis tests during the preliminary statistical phase.

Preliminary analyses were conducted to examine potential gender-related biases, which were subsequently ruled out. No significant main or interaction effects involving gender emerged; therefore, this variable was not included in the following analyses.

## 3 Results

### 3.1 EEG trend analysis on all 56 segments

#### 3.1.1 Delta band

**FIGURE 2:**
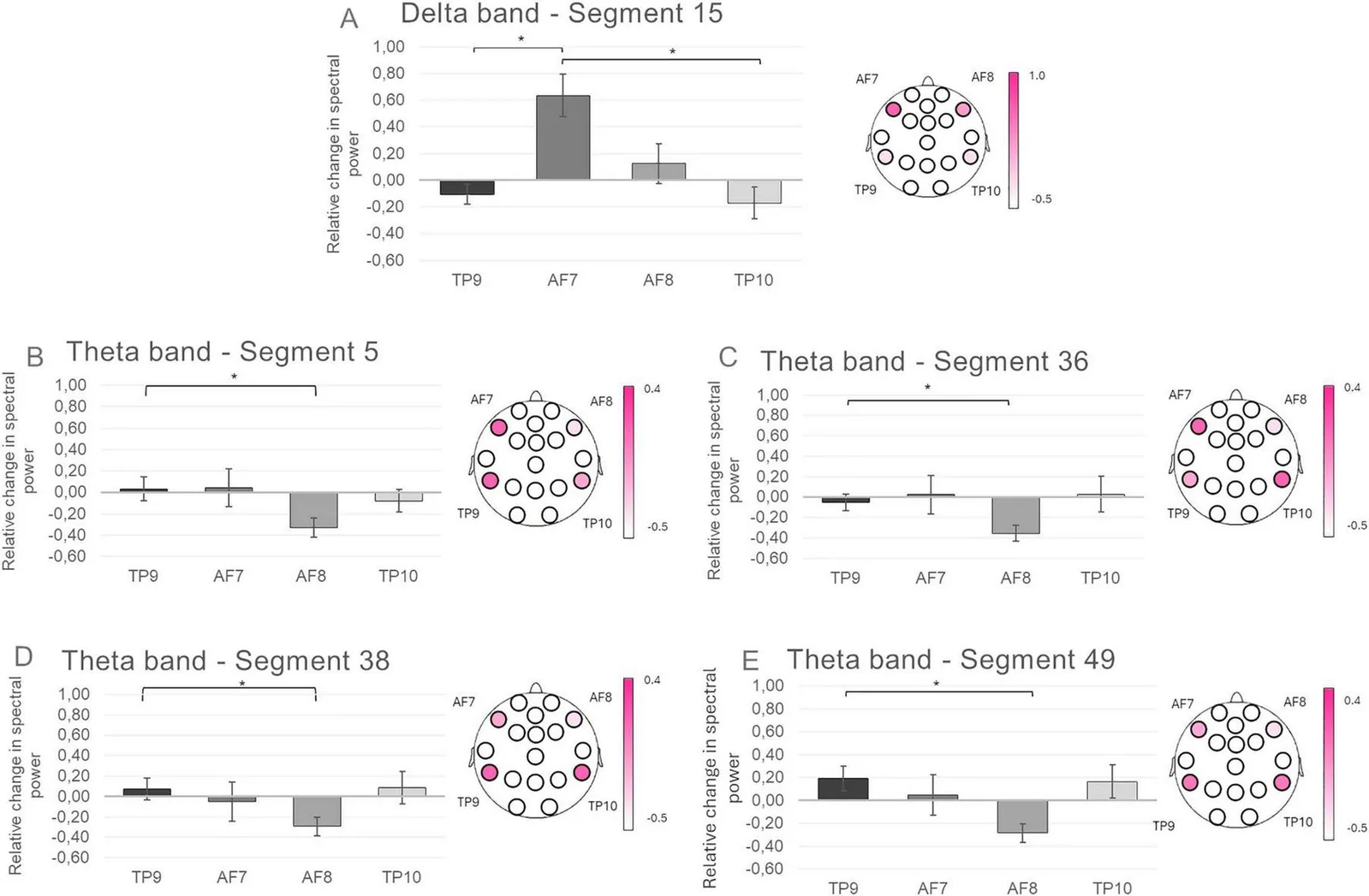
**(A–E)** EEG trend analysis on all 56 segments: The bar charts display the significantly greater delta power observed in AF7 compared to TP9 and TP10 for Segment 15 **(A)** and grater theta power in TP9 compared to AF8 for **(B)** Segment 5; **(C)** Segment 36; **(D)** Segment 38; and **(E)** Segment 49. Values represent relative change in spectral power (baseline normalized). Positive values indicate an increase relative to baseline, while negative values indicate a decrease. For each segment, the more intense color in the rendering of the head (on the left) represents the increase in EEG power at the specific EEG electrode. Error bars represent the mean ± standard error and stars (*) denote statistically significant differences, with *p* ≤ 0.05.

A significant interaction effect was found for Electrodes × Segment (*F* [165,2145] = 1.318, *p* = 0.006, *η*^2^_*p*_ = 0.116). Pairwise comparisons revealed higher delta activity for segment 15 in AF7 compared to TP9 (*p* = 0.005) and to TP10 (*p* = 0.014) [EM_*AF*7_ = 0.635, SE 0.159, 95% CI (0.280, 0.990); EM_*TP*9_ = −0.106, SE 0.076, 95% CI (−0.275, 0.064); EM_*TP*10_ = −0.172, SE 0.119, 95% CI (−0.437, 0.094)] (Figure 2A).

#### 3.1.2 Theta band

A significant interaction effect was found for Electrodes × Segment (*F* [165,2145] = 1.574, *p* < 0.001, η^2^_*p*_ = 0.116). Pairwise comparisons revealed higher theta activity in TP9 compared to AF8 for segment 5 (*p* = 0.027) [EM_*TP*9_ = 0.031, SE 0.111, 95% CI (−0.211, 0.274); EM_*AF*8_ = −0.329, SE 0.089, 95% CI (−0.523, −0.136)] (Figure 2B), for segment 36 (*p* = 0.022) [EM_*TP*9_ = −0.050, SE 0.082, 95% CI (−0.229, 0.128); EM_*AF*8_ = −0.354, SE 0.081, 95% CI (−0.531, −0.178)] (Figure 2C), for segment 38 (*p* = 0.030) [EM_*TP*9_ = 0.072, SE 0.107, 95% CI (−0.162, 0.306); EM_*AF*8_ = −0.291, SE 0.92, 95% CI (−0.493, −0.90)] (Figure 2D), and for segment 49 (*p* = 0.015) [EM_*TP*9_ = 0.195, SE 0.107, 95% CI (−0.038, 0.427); EM_*AF*8_ = −0.283, SE 0.081, 95% CI (−0.460, −0.107)] (Figure 2E).

No significant results were found for alpha, beta, or gamma bands (all *p* > 0.05).

### 3.2 EEG trend analysis on each character

#### 3.2.1 Theta band

**FIGURE 3:**
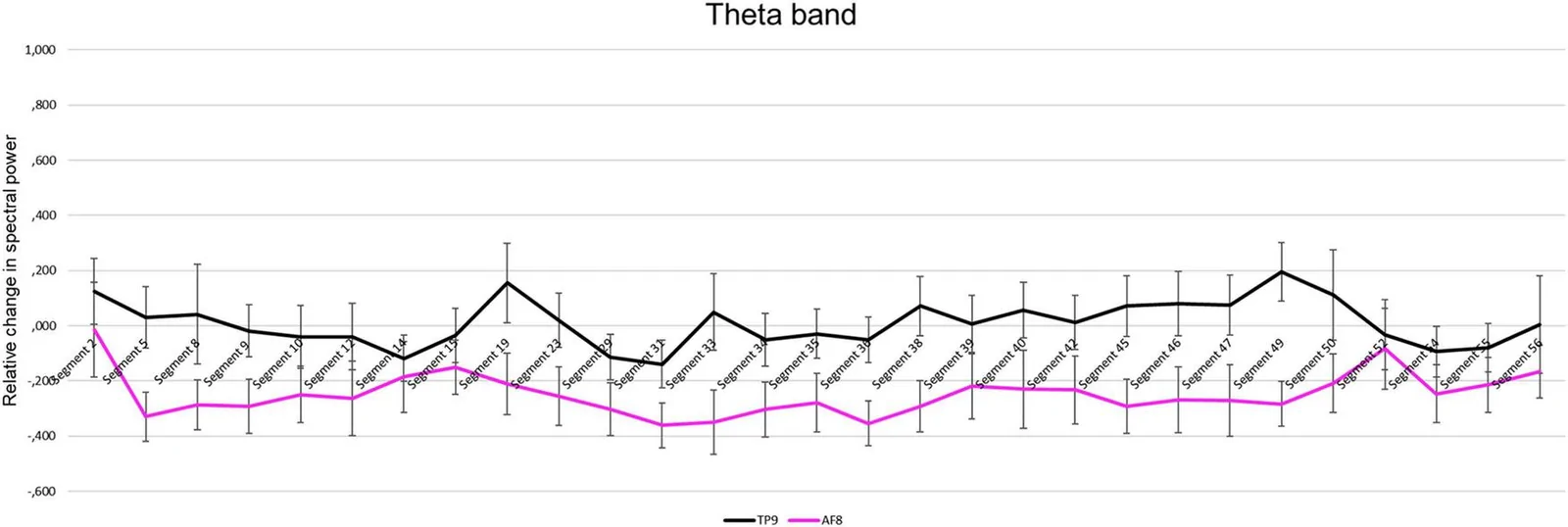
Representative full time-course plot for theta band activity (relative change in spectral power) in TP9 and AF8 for all segments. Error bars represent the mean ± standard error.

For Sophie-related segments (*n* = 29), a significant Electrode × Segment effect was observed in the theta band (*F* [84,1092] = 1.469, *p* = 0.005, η^2^_*p*_ = 0.109): higher TP9 activity compared to AF8 during segment 5 (*p* = 0.027) [EM_*TP*9_ = 0.031, SE 0.111, 95% CI (−0.211, 0.274); EM_*AF*8_ = −0.329, SE 0.089, 95% CI (−0.523, −0.136)], segment 36 (*p* = 0.022) [EM_*TP*9_ = −0.050, SE 0.082, 95% CI (−0.229, 0.128); EM_*AF*8_ = −0.354, SE 0.081, 95% CI (−0.531, −0.178)], segment 38 (*p* = 0.030) [EM_*TP*9_ = 0.072, SE 0.107, 95% CI (−0.162, 0.306); EM_*AF*8_ = −0.291, SE 0.92, 95% CI (−0.493, −0.90)], and for segment 49 (*p* = 0.015) [EM_*TP*9_ = 0.195, SE 0.107, 95% CI (−0.038, 0.427); EM_*AF*8_ = −0.283, SE 0.081, 95% CI (−0.460, −0.107)], confirming the results found in the 56-segment analysis (Figure 3).

For Nazi-related segments (*n* = 18), no significant effects were found for any frequency band (all *p* > 0.05). No other significant effects were found (all *p* > 0.05).

### 3.3 EEG valence analysis

#### 3.3.1 Gamma band

**FIGURE 4:**
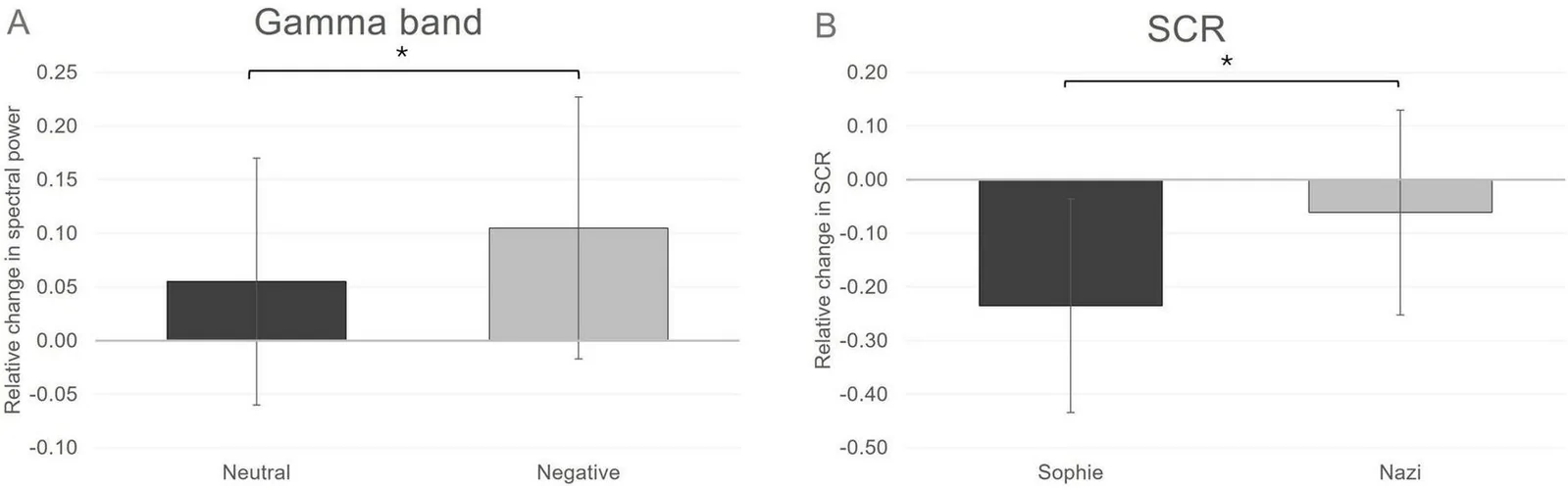
**(A,B)** EEG valence and autonomic analysis: **(A)** The bar charts display the significantly greater gamma power observed in Negative segments compared Neutral segments. Values represent relative change in spectral power (baseline normalized). Positive values indicate an increase relative to baseline, while negative values indicate a decrease. Error bars represent the mean ± standard error. **(B)** The bar charts display the significantly greater SCL values observed in Nazi compared to Sophie. Values represent relative change in SCL (baseline normalized). Positive values indicate an increase relative to baseline, while negative values indicate a decrease. Error bars represent the mean ± standard error and stars (*) denote statistically significant differences, with *p* ≤ 0.05.

For Sophie’s segments, a main effect of Emotion was found in the gamma band (*F* [1,14] = 7.017, *p* = 0.019, *η*^2^_*p*_ = 0.002), with higher gamma activity during negative compared to neutral segments [EM_*negative*_ = 0.105, SE 0.122, 95% CI (−0.157, 0.367); EM_*neutral*_ = −0.055, SE 0.115, 95% CI (−0.192, 0.302)] (Figure 4A).

For both Sophie and Nazi, no significant effects were observed for delta, theta, alpha, or beta bands (all *p* > 0.05).

### 3.4 Autonomic analysis

A significant main effect was found in the within-subject factor Characters (*F* [1,6] = 6.685, *p* = 0.041, *η*^2^_*p*_ = 0.031], with higher levels of SCR for the Nazi character compared to Sophie [EM_*Nazi*_ = −0.061, SE 0.191, 95% CI (−0.528, 0.406); EM_*Sophie*_ = −0.235, SE 0.199, 95% CI (−0.722, 0.252)] (Figure 4B). For all other autonomic indices (SCL, HR and HRV) no significant effect was found (all *p* > 0.05).

## 4 Discussion

The current study investigated neurophysiological and autonomic responses to a highly emotionally charged and morally paradoxical scenario, exemplified through a key scene from Sophie’s Choice. By integrating EEG spectral analysis and autonomic indices, this research explored the dynamic interplay between emotional salience, moral reasoning, and physiological arousal during exposure to a DB scenario.

The EEG trend analysis on all 56 segments revealed significant modulations in both delta and theta band activity. Delta activity increased in the left frontal region (AF7) during segment 15, when Sophie, holding her young daughter, is confronted with the Nazi officer, with evident concern but still uncertain about his intentions. This moment is marked by heightened narrative tension and uncertainty regarding the unfolding situation. Delta enhancement in the left frontal region may reflect processes related to anticipatory cognitive control and emotional regulation (Nobre and van Ede, 2018, Gomez-Ramirez et al., 2011). More general, activity in left frontal areas has been linked with top-down cognitive control processes, which are engaged to extract, evaluate, and integrate salient information in ambiguous contexts (Nobre and van Ede, 2018; Cervantes Constantino et al., 2021). While frontal delta activity has been associated with anticipatory cognitive control in previous literature, the present effect was limited to a single segment and was not observed consistently across similar conditions. A possible interpretation for this result could be framed within the dual-process models of decision-making (Kahneman, 2011). The emotional ambiguity of the scene could easily activate System 1, generating an immediate and downward emotional response to the perceived threat, aroused by the visible fear of Sophie and her daughter. Simultaneously, System 2 might initiate a slower, top-down regulatory process to cognitively evaluate the situation and prepare for an appropriate response. Delta oscillations have been proposed to be involved in the synchronization of large-scale neural networks, enabling communication between frontal regions and areas involved in emotional processing, such as the amygdala and insula (Seeley et al., 2007; Raichle, 2015). Therefore, this finding does not provide systematic support for Hypothesis 1. Rather than reflecting a generalizable response to a clearly defined condition, the observed delta modulation may be related to a specific moment of narrative uncertainty requiring emotional regulation. This evidence should be interpreted cautiously and within the exploratory scope of the study.

An increase in theta activity in the left temporo-parietal area (TP9) emerged during segments where Sophie’s distress was most visible (segments 5, 36, 38, and 49) in both trend analyses. Segment 5 depicts Sophie’s anxious gaze and her quick, fearful response to the Nazi’s questions; in segment 36 her anguished expression dominates the scene following the Nazi’s demand to choose between her children. In segment 38 Sophie’s disbelief and sadness become more pronounced accompanied by her daughter’s fear, while segment 49 represents the culmination of Sophie’s emotional breakdown, marked by her tearful expression and strong distress. Left temporo-parietal regions have been associated with social and emotional processing, including perspective-taking and empathy (Saxe and Wexler, 2005). The selective increase of theta oscillations at temporo-parietal electrode site (TP9) may reflect processes associated with empathic resonance with the narrative, i.e., with Sophie’s suffering rather than reacting to the officer. Crucially, the increase in theta activity at TP9 was accompanied in all segments by a simultaneous decrease at right prefrontal site (AF8). This inverse pattern might suggest neurophysiological modulation reflecting the dilemma’s core conflict. While the TP9 theta increase may indicate intensified empathic resonance with the victim’s distress, the AF8 theta decrease may indicate that deliberative cognitive control processes, associated with the right PFC (Greene et al., 2001, 2004), were temporarily disengaged and modulated by the intense emotional resonance. Overall, this interpretation aligns with theoretical perspectives on moral salience (Decety and Lamm, 2007), which suggest that victim-perpetrator dynamics elicit stronger emotional and moral responses due to their ethical significance. Furthermore, these findings extend prior evidence that theta oscillations mediate social understanding and moral reasoning (Billeke et al., 2014; Cavanagh and Frank, 2014; van Noordt et al., 2015; Tendler and Wagner, 2015). These findings suggest that viewers may have engaged with the dilemma not only at a cognitive level, but also through processes related to emotional resonance with Sophie’s distress.

Additionally, the valence analysis revealed that gamma activity was significantly higher during negative compared with neutral segments depicting Sophie’s character. Previous studies have demonstrated that gamma activity intensifies during emotionally salient experiences, reflecting the brain’s need to allocate resources to particularly relevant and impactful stimuli (Luo et al., 2014; Knyazev et al., 2016). These results may be interpreted in light of frameworks related to affective mirroring, although no direct inference regarding gamma oscillation and MNS activation can be drawn from the present data. The MSN, implicated in the mirroring of observed emotions and actions, enabling individuals to vicariously experience the emotional states of others (Cattaneo and Rizzolatti, 2009; Lamm and Majdandžić, 2015). When external observers are exposed to Sophie’s negative emotional expressions – her despair, sadness, and visible distress – gamma oscillations may reflect increased neural activity associated with the processing of emotionally salient stimuli and integrative processes. Perhaps the need for this integration processing could be due to the affective mirroring and emotional resonance with Sophie’s condition. On the other hand, the absence of gamma enhanced gamma activity for the Nazi-related segments suggests that empathic resonance was directed toward the victim rather than the aggressor. Indeed, research in moral neuroscience has shown that empathic resonance and emotional salience are more readily triggered by the suffering of innocent individuals than by the actions of aggressors, even when those actions are morally reprehensible (Decety and Lamm, 2007).

Autonomic results complemented EEG findings by showing increased SCR during Nazi-related segments. Externalized sources of threat, such as aggressive or hostile behaviors, are typically associated with increased autonomic arousal, often linked to defensive responses (i.e., immediate fight-or-flight response), which is mediated by the sympathetic nervous system and reflected in transient increases in SCR (Critchley, 2002). The Nazi-related segments likely engaged this reactive arousal system, as the observer’s attention was drawn to the overtly threatening actions of the aggressor. Studies have shown that stimuli involving clear externalized threats – such as verbal or physical aggression – are processed more rapidly and robustly by the salience network compared to stimuli involving more subtle or internally focused emotional cues (Gan et al., 2016; Klasen et al., 2019; Puiu et al., 2020). This difference likely accounts for the lower SCR levels during Sophie’s scenes, as her internal suffering might have elicited a more cognitive-emotional response rather than a purely physiological arousal driven by externalized salience. Interestingly, other autonomic indices, such as HR, HRV, and SCL, did not show significant differences between the two conditions. This result highlights the transient and context-specific nature of SCR as a marker of emotional arousal (Laine et al., 2009). Given the reduced sample size for autonomic indices, particularly SCR, these findings should be interpreted as tentative and requiring replication in larger samples.

To conclude, the current study offers valuable preliminary insights into the interplay between neurophysiological and autonomic responses when individuals engage with emotionally intense and morally complex narratives, exemplified through the Sophie’s Choice scenario.

Several limitations must be acknowledged. First, the sample size was small (*N* = 16), limiting generalizability. Future studies should include larger and more diverse samples. Second, only one cinematic stimulus was used, raising concerns about stimulus specificity. Including multiple films or narrative contexts would allow stronger conclusions. Third, EEG data were recorded using a low-density wearable device, which limited spatial resolution. The present results are therefore interpreted in terms of general regional activity (e.g., “left frontal” and “left temporo-parietal” regions). Future research using high-density EEG or fMRI could further and more accurately test the involvement of specific cortical structures such as the DLPFC and TPJ. Although the present analysis focused on emotional valence (neutral vs. negative segments), differences in emotional intensity could also play a role. However, the scene unfolds within a relatively constant setting, with stable lighting, camera framing, and composition, suggesting that these cinematic features are unlikely to have substantially influenced the perceived intensity across segments. Future research could systematically investigate how such cinematic and emotional parameters modulate neurophysiological responses.

Finally, autonomic measures were limited to SCL, SCR, HR, and HRV; future work could integrate additional measures such as facial electromyography or pupil dilation to capture finer-grained emotional responses, as well as including subjective measurements of the viewer’s emotional and moral experience. Moreover, future studies using larger samples could explore how gender and parental status influence emotional and cognitive reactions, given the maternal themes evoked by the scene.

## 5 Conclusion

This study contributes to understanding the neurophysiological responses elicited by morally paradoxical film scenes. Delta activity in the left frontal region may be related to anticipatory control processes during a specific narrative ambiguous segments (rather than be generally associated with segments valence), while theta activity in the temporo-parietal areas may reflect processes associated with empathic engagement with the victim’s suffering. Gamma oscillations may reflect emotional resonance with negative content, and SCR increases might suggest autonomic arousal to external threat salience. Together, these findings highlight the complementary contributions of neural and autonomic systems in processing double binds, and support the use of cinema as an ecological method for studying the neurophysiology of moral and emotional dilemmas. This pilot study provides preliminary insights into the neurophysiological correlates of moral and emotional processing in complex narrative contexts.

## Data Availability

The data presented in this study are available on request from the corresponding author due to ethical reasons for sensitive personal data protection (requests will be evaluated according to the GDPR - Reg. UE 2016/679 and its ethical guidelines). Requests to access the datasets should be directed to FC, flavia.ciminaghi@unicatt.it.
